# Optimization and Analysis of Refill Friction Stir Spot Welding (RFSSW) Parameters of Dissimilar Aluminum Alloy Joints by FE and ANN Methods

**DOI:** 10.3390/ma17184586

**Published:** 2024-09-18

**Authors:** Dan Cătălin Bîrsan, Florin Susac, Virgil Gabriel Teodor

**Affiliations:** Department of Manufacturing Engineering, Faculty of Engineering, “Dunărea de Jos” University of Galați, Str. Domnească No. 111, 800201 Galati, Romania; dan.birsan@ugal.ro (D.C.B.); florin.susac@ugal.ro (F.S.)

**Keywords:** RFSSW, artificial neural network, numerical simulation, aluminum alloy, dissimilar

## Abstract

The quality of the refill friction stir spot welding (RFSSW) process is heavily dependent on the selected welding parameters that influence the resultant joint characteristics. Thermomechanical phenomena integral to the process were investigated using finite element (FE) analysis on two dissimilar materials. This FE analysis was subsequently validated through controlled experiments to ensure reliability. An artificial neural network (ANN) was employed to create a neural model based on an experimental setup involving 120 different sets of welding parameters. The parameters adjusted in the experimental plan included pin penetration depth, rotational speed, retention time, and positioning relative to material hardness. To assess the neural model’s accuracy, outputs such as maximum temperature and normal stress at the end of the welding process were analyzed and validated by six data sets selected for their uniform distribution across the training domain.

## 1. Introduction

More than 20 years ago, a German company [1] introduced, for the first time, a refill friction stir spot welding process (RFSSW) as a derivative welding process of the spot friction welding process (FSSW). The key distinction lies in the fact that when using RFSSW, no hole remains in the sheet after the welding of the joint. The rotary tool comprises an external stationary clamping ring, a sleeve, and a rotating pin. The primary function of the clamping ring is to secure the two metal sheets in place. The sleeve is capable of vertical movement independent of the pin, which has the ability to both rotate and translate vertically.

**Figure 1:**
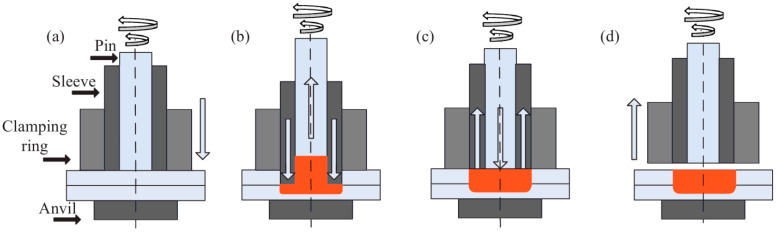
The four steps of the RFSSW process: touchdown and preheating (**a**), plunging (**b**), refilling (**c**), and retreating (**d**) [2].

The scientific literature presents this process as structured into four stages: touchdown and preheating, plunging, refilling, and retreating. In Stage 1, touchdown and preheating, the welding head secures the two metal sheets using the clamping ring and sleeve, while the rotating pin generates localized heat. In Stage 2, plunging, the sleeve moves downward in coordination with the pin retracting, causing material redistribution. Stage 3, refilling, involves the synchronized downward movement of the rotating pin as the sleeve translates in the opposite direction. In the fourth stage, the welding head assembly is withdrawn [2] (see Figure 1).

The RFSSW welding technique is well suited for successful application in many industries due to its advantages such as high-quality joints, lower residual stresses than in the case of joints obtained by fusion welding, and the possibility of making joints from dissimilar materials. Moreover, this technology is environmentally friendly, does not require material addition, and presents a lack of gas emissions. Therefore, RFSSW is conducive to automation, allowing for seamless integration into existing manufacturing processes. This automation capability leads to increased productivity.

To understand the phenomena occurring while using the RFSSW welding technique, several experiments and numerical modeling were recently conducted by many researchers.

For example, Harachai et al. [3] investigated the FSW process optimization of dissimilar aluminum alloys. The effect of process parameters on the quality of butt joints was analyzed. The response surface methodology (RSM) and analysis of variance (ANOVA) were used to analyze the effect of each parameter on the mechanical properties of the materials. It was concluded that the process parameters have a significant influence on the tensile strength of the joints, and the optimum combination of parameters was identified for the best tensile strength of 190 MPa and 80 HV hardness.

Kubit et al. [4] conducted a study to evaluate the impact of welding parameters on the quality of welded joints made of AA7075-T6 material. The results showed that an improper selection of welding process parameters led to the appearance of major defects.

Andres et al. [5] carried out research on overlap welding using the RFSSW process. They varied the rotational speed and the penetration depth of the tool sleeve in order to examine the influence of the welding process-specific parameters on the characteristics of lap joints.

Research carried out by Ziółkowski et al. [6] in the field of dissimilar material joints examine the mechanical properties of butt joints between steel and aluminum alloys. They analyzed the relationship between these properties and the changes that appear in the structure of the material and are caused by welding. Following macro- and microstructural investigations, they identified the characteristic zones of FSW joints, with microstructure analysis focusing on the material interface line in the root zone. The negative impact on the joint quality was demonstrated through deformation analysis using digital image correlation performed during the test of traction.

Shen et al. [7] studied the RFSSW welding process of dissimilar aluminum alloys, performed using a profiled tool. The obtained results reconfirmed that the welding procedure involved a combination of reverse extrusion and forging.

By varying the parameters of the FSW welding process, Kumar et al. [8] aimed to optimize the rotation speed, welding speed, and tool inclination angle to obtain high tensile strength and appropriate hardness in welded joints.

Zou et al. [9] conducted a study to investigate the influence of rotational speed and immersion depth on the macro/microstructures and mechanical properties of dissimilar aluminum alloy joints. The study showed that changes in rotational speed have no significant impact on the appearance of hook-type defects, while an increase in the penetration depth leads to a significant increase in the height of these defects. 

An experimental investigation of the friction stir spot welding (FSSW) technique for push-joining thin sheets of AA6061-T6 was carried out by D’Urso et al. [10]. Variations in the tool rotation speed were applied and the experimental validation confirmed the findings from the numerical analysis.

Mortello et al. [11] aimed to evaluate the experimental investigation of the material characteristics in friction stir-welded (FSW) joints made of different aluminum and steel. This finding implies that progressively changing thermal conditions, dissipation characteristics, and improved metal bonding as the tool advances along the weld line may contribute to improved fatigue performance in the second part of the weld.

A comprehensive thermomechanical finite element model specifically for reflow friction spot welding (RFSSW) of aluminum alloy 6061-T6 was designed by Muci-Küchler et al. [12]. The accuracy of the finite element analysis was confirmed by aligning its predictions with empirical data from experimental observations.

Using finite elements software, Cao et al. [13] explored material flow characteristics during friction stir spot welding (FSSW) of AA6061 sheets. By adjusting welding parameters, their study aimed to analyze the formation of the “hook” defect and onion ring structure at the edge of the sleeve.

The impact of processing parameters on the corrosion properties of dissimilar friction stir welds between aluminum and copper was examined by Akinlabi et al. [14]. The study identified optimal conditions for minimizing the corrosion rate at a rotation speed of 950 rpm and a feed rate of 300 mm/min, resulting in a weld with low heat input.

Using a machine learning algorithm, Effertz et al. [15] sought to optimize the parameters of the welding process in frictional reloading points. Their study showed that the model was highly dependent on the quadratic nature of the evaluated parameters, with both weld duration and rotation speed having a negative impact on the ultimate layer shear strength (ULSF). 

The influence of jointing time on the quality of RFSSW joints was investigated by Donga et al. [16]. The study provided valuable information on optimizing the RFSSW process for Al/Mg alloy joints.

Schmal et al. [17] examine the quality, application, and testing of refill friction stir spot welding in high strength aluminum alloys, offering advanced strategies for industrial use and future research directions.

A comprehensive thermomechanical numerical model tailored to the refill friction stir spot welding (RFSSW) of 7075-T6 aluminum alloy sheets was created by Kubit et al. [18]. Validated through finite element analysis against experimental results, the model accurately predicted the material flow observed in experimentally produced joints. 

Silva et al. [19] conducted a study to optimize the process parameters for refill friction stir spot welding (RFSSW) of AA6082-T6 aluminum alloy. The study highlighted the crucial role of plunge depth in shaping the development and morphology of the hook defect, showing that the fracture behavior of the welds was predominantly influenced by the characteristics of this defect.

An extensive analysis and classification of defects in aluminum joints created using refill friction stir spot welding (RFSSW) technology were conducted by Adamus et al. [20]. The research identifies various failure modes and illustrates how defects develop at the sleeve–plunger interface under worsening process conditions.

A novel technique that integrates hybrid refill friction stir spot welding (RFSSW) with ultrasonic oscillation for joining 5A06 aluminum alloy was introduced by Liu et al. [21]. The study focused on optimizing the process parameters for ultrasonic-assisted RFSSW.

A 2D axisymmetric model for the rotary friction stir spot welding (RFFSW) process was developed by Berger et al. [22,23], effectively capturing its core physics. The study suggests that, with enhanced flow stress data, the model could become a valuable tool for optimizing the RFFSW process, aiding in determining the optimal welding time while ensuring the appropriate temperature conditions for strong bonding.

A novel technique known as scrubbing refill friction stir spot welding (Sc-RFSSW) was introduced by Takeoka et al. [24]. Experimental results demonstrated that increasing the maximum load of the welded joint was achieved by reducing the tool’s penetration depth into the lower plate, ensuring it only engaged the interface. The optimized Sc-RFSSW parameters consistently resulted in stable nugget pullout during both tensile shear and cross-tension tests.

Considering the aspects presented previously in this paper, creating a predictive model based on a neural network capable of predicting the quality of welded joints is suggested. The prediction will be made by analyzing the respective input variables: the properties of the materials used, the geometry of the tool, and the process conditions. This model can identify the optimal process parameters to achieve the desired weld quality while minimizing defects and reducing machining time.

In the field of analysis and simulation of friction stir welding (RFSSW) processes, combinations of optimization algorithms and artificial neural networks are frequently used to improve the performance of predictions made by neural networks.

The main reasons for using ANN analysis in RFSSW include the following. Parameter optimization: ANN helps select the appropriate process parameters for specific welding tasks. By training the network on a data set containing various combinations of process parameters and their outputs, ANN can indicate the optimal sets of parameters to achieve the desired quality attributes. This approach saves time and resources by guiding operators in choosing the appropriate parameters, eliminating the need for extensive trial and error.

A hybrid approach is usually used [25,26,27,28,29], combining the capabilities of an optimization algorithm with the learning power of a neural network. Basically, the optimization algorithm is used to adjust the learning parameters of the neural network, achieving faster convergence, avoiding local minima, and minimizing global error. Among these methods, the following can be mentioned:

The EHGWOA—BPNN (enhanced hybrid gray wolf optimizer algorithm—backpropagation neural network) method [25]. EHGWOA it is used to adjust parameters, such as initial weights and learning rates, to speed up convergence and avoid local minima. The presented algorithm combines with BPNN (backpropagation neural network) to minimize the global error.

The IPSO-RBF method (improved particle swarm optimization—radial basis function) [26], which combines the IPSO algorithm, is based on the social behavior of birds, with a neural network of the RBF (radial basis function) type, which uses radial functions as activation functions.

The BPNN—GA (backpropagation neural network—genetic algorithm) method [27], in which the genetic algorithm is used to find an optimal parameter setting for the backpropagation neural network, allows for the model to achieve better performance and avoid stagnation in local minima.

The PIO—ANN (pigeon-inspired optimization—artificial neural network) method [28] based on the combination of the PIO algorithm, which is later used to optimize the parameters of the neural network.

RBFNN—GWO (radial basis function neural network—gray wolf optimizer) method [29], which uses radial functions for activation and whose parameters are optimized based on the GWO (gray wolf optimizer) algorithm, allows for the model to perform better in solving complex problems such as classification and regression.

Weld quality prediction: One of the main advantages of using ANN is its ability to accurately predict the final weld characteristics such as strength and hardness. By analyzing and learning from the historical process data, ANN can suggest parameter adjustments to minimize defects and ensure consistent and superior weld quality. This is essential for critical applications where reliability and welding performance are of paramount importance.

Improving process efficiency: Through its ability to model complex relationships between different process parameters, ANN allows for their optimization in a precise and efficient way. Thus, optimal parameter settings can be determined quickly and accurately, resulting in a reduction in experimentation and adjustment time, saving valuable resources and increasing overall productivity.

Improved control and monitoring: Integrating ANN into RFSSW systems enables real-time monitoring of the welding process. By continuously analyzing sensor data, ANN can quickly detect anomalies and variations in process parameters, providing immediate feedback for adjustments. This not only prevents defects and reduces the scrap rate but also allows for quick and precise interventions, keeping the welding quality at an optimal level.

Automation and optimization of operations: The power of machine learning provided by ANN enables the extraction of valuable information from large volumes of data, thereby facilitating the optimization and automation of RFSSW operations. By using this information, processes can be automatically adjusted and adapted to respond to variations in production conditions, thus ensuring high flexibility and adaptability.

Reduction of costs and production time: Optimization of process parameters and accurate prediction of weld quality leads to a significant reduction of costs associated with materials and production time. By avoiding defects and the need for repairs, resources are saved, and process profitability is improved.

Improving diagnostic and maintenance capabilities: ANNs are able to identify patterns and trends in historical and real-time data, enabling early diagnosis of problems such as tool wear or material inconsistencies. This facilitates predictive maintenance and prompt intervention, preventing unplanned outages and extending equipment life.

Overall, the use of ANN analysis in RFSSW leads to smarter and more efficient manufacturing. Harnessing the power of machine learning and advanced data analysis not only improves the quality and consistency of welds but also enables more precise and adaptive management of the entire production process, paving the way for the development of advanced welding technologies.

The novelty of this work consists in the fact that by validating the experimental results with data obtained by Simufact Forming simulation, we have demonstrated the ability of this software to achieve a sufficiently accurate prediction for this type of application. This has direct implications on material and energy consumption in the case of optimization of process parameters. Last but not least, the possibility of using the trained neural network in order to obtain the quality characteristics of the process opens up possibilities related to the estimation of the results in case one of the process parameter sizes is imposed for various reasons. 

Building upon the previous analysis, this paper suggests the creation of a neural network-based predictive model capable of accurately forecasting weld joint quality based on input variables such as the properties of the materials used, tool geometry, and process conditions. This model can identify optimal process parameters to achieve the desired weld quality while minimizing defects and reducing processing time. Artificial neural network (ANN) analysis is widely applied in refill friction stir welding (RFSSW) due to its numerous benefits. Key reasons for utilizing ANN analysis in RFSSW include the following. Parameter optimization: ANNs assist in selecting suitable process parameters for specific welding tasks. By training the network on a data set containing various combinations of process parameters and their outcomes, the ANN can indicate optimal parameter sets to achieve the desired quality attributes. This approach conserves time and resources by guiding operators in choosing appropriate parameters, thus eliminating the need for extensive trial and error.

## 2. Materials and Methods

The study aimed to evaluate the effect of welding process parameters on the thermal distribution within the weld zone and the mechanical properties of RFSSW welded joints. Experiments were conducted on two aluminum alloy sheets, 6061-T6 and 7075-T6, both 1 mm thick. Table 1 shows the chemical compositions of these two alloys. The sheets, measuring 100 × 100 × 1 mm (L × W × T), were superposed and welded using a cylindrical tool mounted on a welding machine. The welding tool consisted of three main components: a blank-holder with a 24 mm diameter, a sleeve with a 9 mm diameter, and a pin with a 5.5 mm diameter.

### 2.1. Methodology of the Welding Process

**Figure 2:**
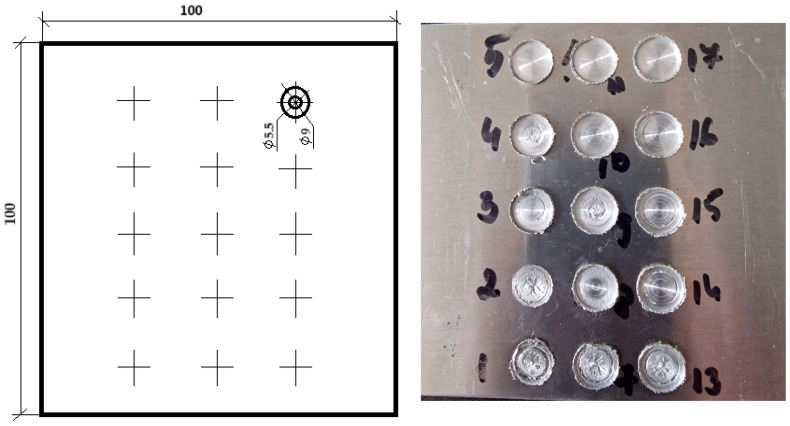
Shape and dimensions (in mm) of the welding plates.

**Figure 3:**
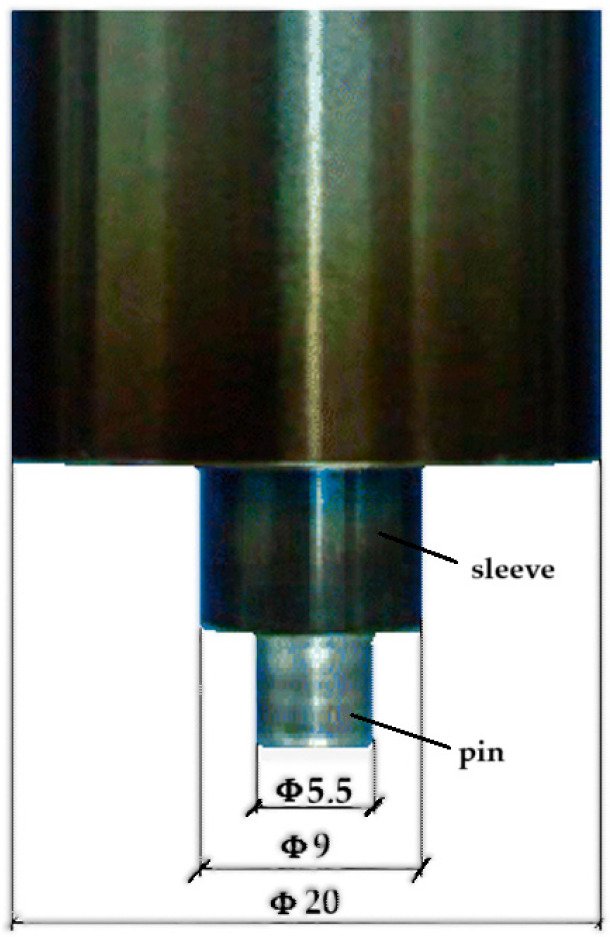
Shape and dimensions (in mm) of the tool.

Initially, experimental tests were performed by varying the welding process parameters. During the welding process, temperature was measured at certain points in the welding area (as presented in Section 2.2). The measured temperatures were subsequently used to validate the finite element model that was developed to simulate the welding process. The comparison between experimental and numerical data is presented in paper for one of the studied cases. After the validation of the numerical model, the maximum temperature and maximum equivalent stress, respectively, the temperature and equivalent stress at the end of the welding process, were collected for various combinations of the process parameters. These combinations were used as input data for training a neural network (JustNN, version 4.a) to predict the temperature and equivalent stress corresponding to new parameter combinations. The neural model validation was performed using parameter combinations obtained from the simulation, not used in the neural network training. Figure 2 shows the dimensions of the parts to be welded and different welding points for different parameters (starting with point 1, made at a rotation speed of 2400 rpm and penetration depth of 0.85 mm and ending with point 17–3200 rpm and 0.95 mm) and Figure 3 shows the shape and dimensions of the tool used during the welding process. The parameters used to realize the joints in Figure 2 can be seen in Table 2. The workpieces were secured to the machining table using clamps. The tool’s rotational speed ranged from 2400 to 3200 rpm in increments of 200 rpm, and the penetration depth varied from 0.6 to 0.9 mm in increments of 0.1 mm. The welding cycle included a plunging time of 1 s and a refilling time of 1 s.

After welding, metallographic analysis was conducted by polishing the samples and examining them microscopically to highlight the size and shape of the material grains.

### 2.2. Temperature Measurement

**Figure 4:**
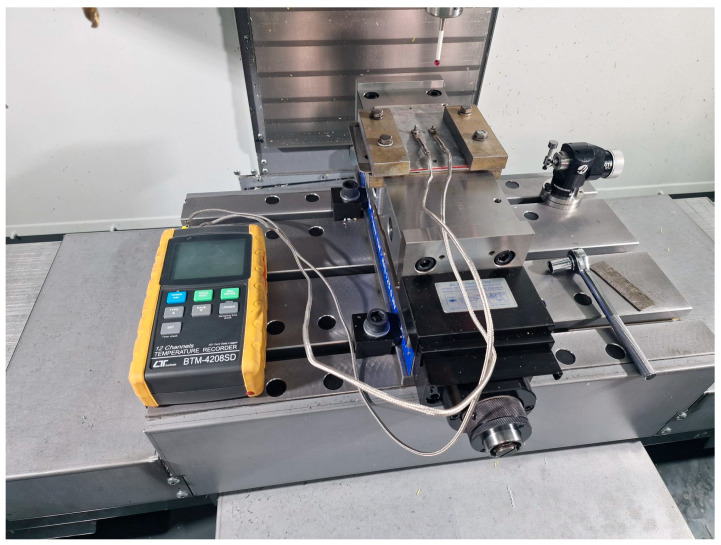
The temperature measurement system.

During the welding process, temperature measurements were performed using two K-type thermocouples symmetrically positioned on the top surface of the top plate, 5 mm away from the center of the pin, as illustrated in Figure 4, and connected to a BTM-4208SD temperature recorder. The temperature data were recorded at 200 ms intervals, and to ensure accuracy, measurements were taken on 20 samples, with the average values obtained being used to validate the finite element analysis.

### 2.3. Finite Element Modeling

Finite element analysis was employed to model and simulate the joining process, with results validated by assessing the temperature distribution in the upper plate during experiments. Using the commercial software package Simufact Forming 16.0 [30] (Simufact Engineering GmbH, Hamburg, Germany) a 2D axisymmetric finite element model was developed to simulate the RFSSW process. This model used a fully coupled thermomechanical approach, allowing simultaneous calculation of temperature and strain at each time increment. The material properties (as can be seen in Table 3) were obtained from the Simufact Forming software database and represent a temperature and strain rate-dependent material model based on the MatiLDa database, a registered trademark of Gesellschaft für metallurgische Technologie-und Softwareentwicklung mbH (Berlin, Germany).

The following equation represents the mathematical model of the material behavior:

(1) $$\sigma_{F}=C_{1}\cdot e^{\left(C_{2}\cdot T\right)}\cdot \varepsilon_{p}^{\left(n_{1}\cdot T+n_{2}\right)}\cdot e^{\left(\frac{l_{1}\cdot T+l_{2}}{\varphi }\right)}\cdot {\dot{\varepsilon }}_{p}^{\left(m_{1}\cdot T+m_{2}\right)}$$

where *T* represents temperature, *ε_p_* denotes plastic strain, ${\dot{\varepsilon }}_{p}$ signifies plastic strain rate, and *C*_1_, *C*_2_, *n*_1_, *n*_2_, *l*_1_, *l*_2_, *m*_1_, *m*_2_ represent the parameters obtained from the experimental data adapted to the plasticity model Equation (1); in Table 4, you can see the values of these parameters for the two materials that were used in the modeling. The method for determining these parameters is not provided by the Simufact Forming software.

**Figure 5:**
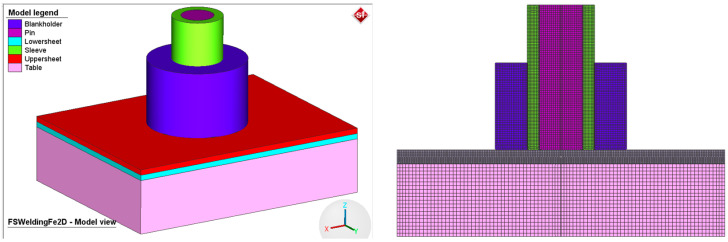
Finite element mesh of the 2D axisymmetric model.

**Figure 6:**
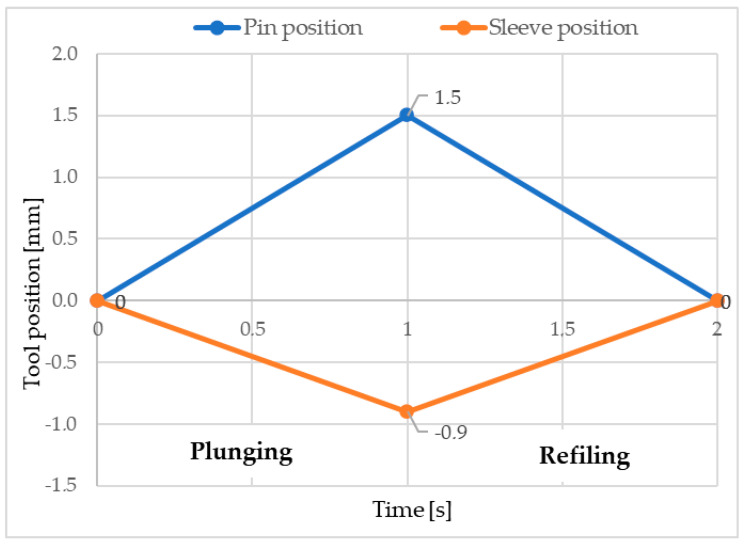
RFSSW process steps considered in the numerical model (0.9 mm depth).

The finite element model consists of the workpieces, the welding tool, and a table, as can be seen in Figure 5. The bottom surface of the bottom plate was fully constrained to prevent displacement in any direction. In order to reflect the real conditions, some simplifications were made in the numerical model without affecting the results: the 2D elements designed for the analysis of 2D axiometric problems, known as Quad (10) in Simufact Forming terminology [30], were used for modeling the finite elements; the pin, sleeve, and snap ring were set as rigid bodies; the rotation speed and tool displacements were set to reproduce the experimental conditions (see Figure 6); an automatic resizing feature was used to regenerate the meshes and continue the simulation with the new mesh configuration. The top and bottom plates were modeled using a total of 2380 elements, the welding tool and support plates were modeled as rigid bodies. All rigid bodies were modeled using 5020 quadric elements called Quad (40) [30].

The equations that have been used to analyze the RFSSW process are as follows:

- Heat transfer equation during the RFSSW process

(2) $$\frac{\partial T}{\partial t}=\frac{\partial }{\partial x}\left(k_{x}\frac{\partial T}{\partial x}\right)+\frac{\partial }{\partial y}\left(k_{y}\frac{\partial T}{\partial y}\right)+\frac{\partial }{\partial z}\left(k_{z}\frac{\partial T}{\partial z}\right)+{\dot{q}}_{p}$$

where *T* is the temperature, ${\dot{q}}_{p}$—the rate of heat generated by the dissipation of plastic energy and *x*, *y*, *z*—the spatial coordinates.

- The equation for calculating the heat generation rate resulting from the dissipation of plastic energy is

(3) $${\dot{q}}_{p}=\tau \cdot \eta \cdot {\dot{\varepsilon }}_{p}$$

where *τ* is the shear stress, *η* is the factor of conversion of mechanical to thermal energy.

- To determine the heat generated by friction between the tool surfaces and the workpiece:

(4) $${\dot{q}}_{f}=\mu \cdot p\cdot \omega$$

where ${\dot{q}}_{f}$ is heat produced by friction, where *μ*—friction coefficient, *p*—contact pressure and *ω*—rotational speed.

- The equation for convective heat dissipation is:

(5) $$q_{c}=h_{f}\left(T_{s}-T_{\infty }\right)$$

where *h_f_* is the convection coefficient (*h_f_* = 50 W/m^2^ °C), *T_s_*—the temperature at the surface of the plates and *T_∞_*—the temperature of the surrounding environment.

- The equation for radiation heat dissipation is:

(6) $$q_{r}=k\cdot \varepsilon_{r}\left(T_{r}^{4}-T_{\infty }^{4}\right)$$

where *κ* is Stefan-Boltzmann constant (*κ* = 5.67 × 10^−8^ W/m^2^ °C), *T_r_*—the absolute temperature of the radiating surface, and *ε_r_*—the emissivity of the radiating surface. 

Equation to calculate the minimum torque required during the plunge phase of the tool is:

(7) $$M=\int_{0}^{R}2\cdot \pi \cdot \mu \cdot F\cdot r^{2}dr$$

where *R* is the radius of the contact surface and *F* is the force.

**Figure 7:**
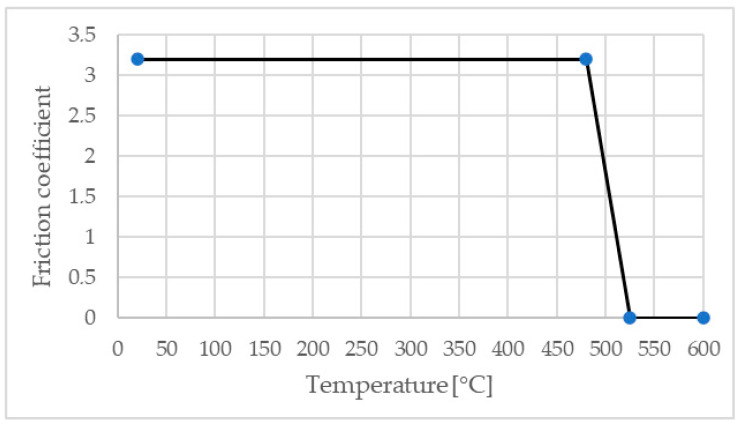
Variation of friction factor vs. temperature.

The variation of the material physico-mechanical properties, including Young’s modulus, Poisson’s ratio, coefficient of thermal expansion, thermal conductivity, and heat capacity due to temperature variations, were incorporated into the material database in the Simufact Forming program [30]. The friction factor was adjusted as a function of temperature (Figure 7), as studied by Zhao et al. [31] and Song et al. [32].

## 3. Results and Discussion

### 3.1. Thermomechanical Results of RFSSW Process

**Figure 8:**
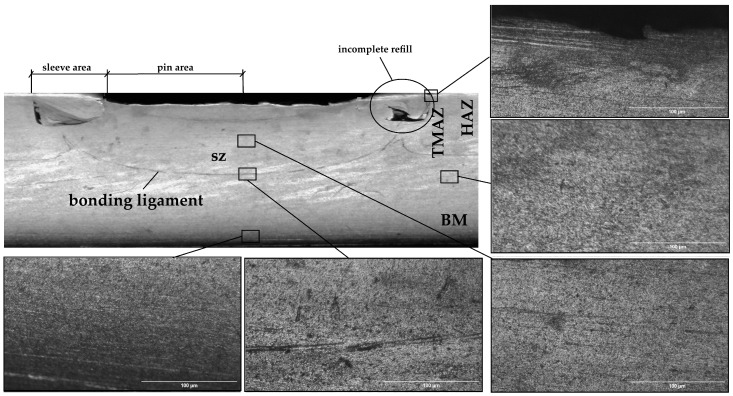
Cross section through the welded joint.

The cross section depicted in Figure 8 shows distinct zones with different structures: the stirring zone (SZ), the thermomechanically affected zone (TMAZ), the heat affected zone (HAZ) and the base material (BM). For the metallographic analysis, the cross section was examined.

The influence of lap interface morphologies on the joint failure load has been extensively studied by several researchers, each highlighting critical aspects of this relationship. Rosendo et al. [33] emphasize that the variations in lap interface morphology, such as the presence o36f voids or incomplete bonding, significantly alter the distribution and magnitude of stresses across the welded area. These variations can lead to stress concentrations that decrease the overall load-bearing capacity of the joint. They further note that the surface smoothness and uniformity of the lap interface are crucial for ensuring an even load transfer, which directly impacts the joint’s ability to withstand higher loads, thereby enhancing the joint failure load.

Similarly, Zhiqing et al. [34] concluded that specific lap interface morphologies, such as the downward-bending hook and the protruding stir zone (SZ), are vital in determining the joint failure load. These features influence stress distribution and bonding strength, with a well-formed lap interface—characterized by minimized defects and optimized morphology—enhancing the effective lap width (ELW) and effective sheet thickness (EST). This optimization leads to improved resistance to shear and tensile loads, ultimately resulting in a higher joint failure load.

Yao et al. [35] also found that the morphology of the lap interface plays a direct role in influencing the joint failure load by affecting structural integrity and load distribution. The presence of hooks or voids within the interface can cause stress concentrations, weakening the joint and reducing its load-bearing capacity. Conversely, a smooth, well-bonded lap interface promotes even stress distribution, enhancing the joint’s ability to sustain higher loads before failure occurs.

#### 3.1.1. Temperature Distribution

As a result of the friction and plastic deformation process in the welded area, an amount of heat is generated which leads to a temperature change in this area.

**Figure 9:**
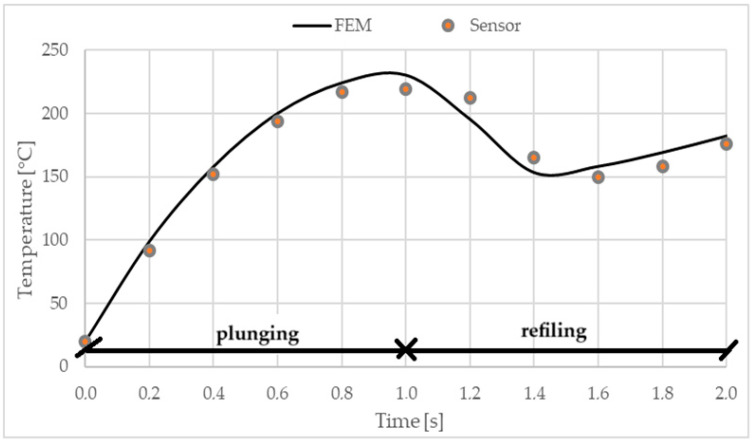
Temperature variation during the welding process at the sensor point and corresponding FEM node.

**Figure 10:**
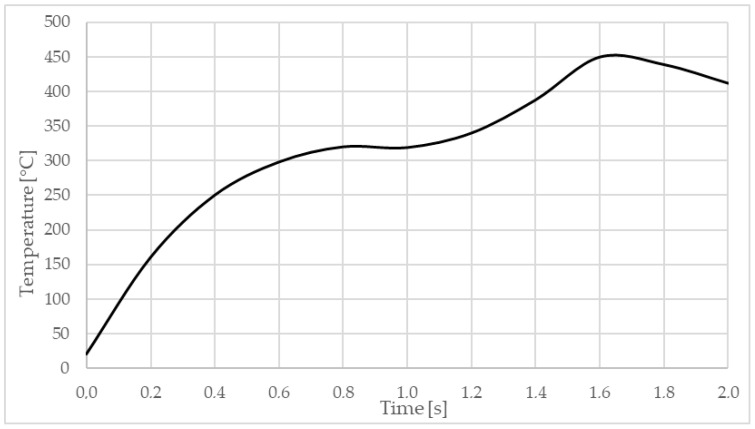
Temperature variation during finite element analysis.

The average temperature values obtained from the sensors mounted on the top plate are compared with those collected from the finite element model at the same points, and these values are plotted as graphs in Figure 9. The initial temperature for the numerical model was set to 20 °C, corresponding to the ambient temperature recorded during the welding process. The rapid rise in temperature occurs in the first stage, which lasts for one second, at the end of which the sensors recorded an average temperature of 230 °C (see Figure 9). The temperature in the weld zone increases rapidly at a rate of 400 °C/s (between 0 and 0.8 s, see Figure 10), with a maximum during the welding process of approximately 450 °C at an instant time of 1.6 s. This maximum temperature is reached when the front edge of the pin meets the inner boundary of the sleeve.

**Figure 11:**
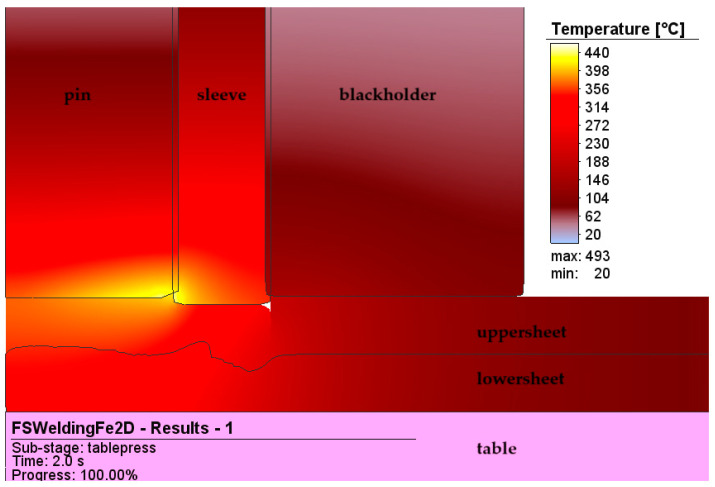
The temperature at the interface of the tool and the workpiece at the welding time corresponding to 2 s.

Figure 11 presents the temperature at the tool/workpiece interface at a welding time that corresponds to 2 s.

**Figure 12:**
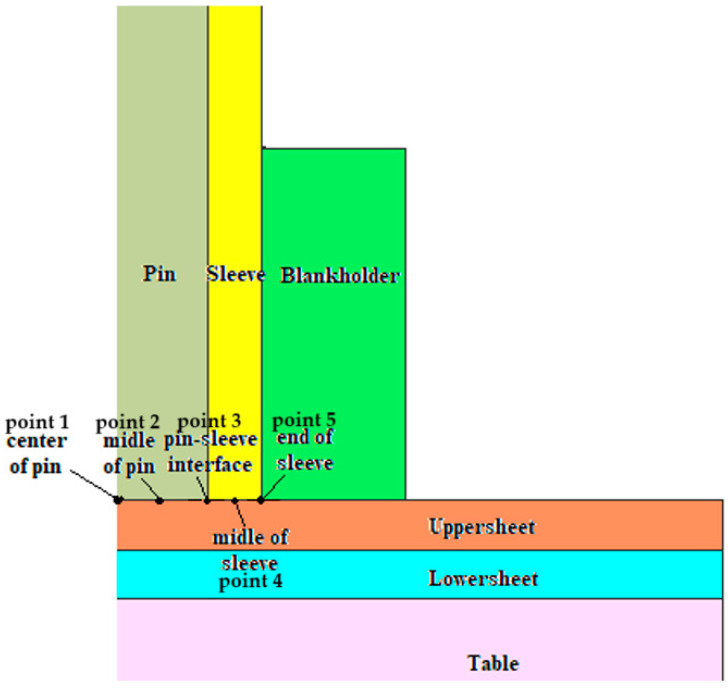
The points selected to illustrate the temperature variation over time.

**Figure 13:**
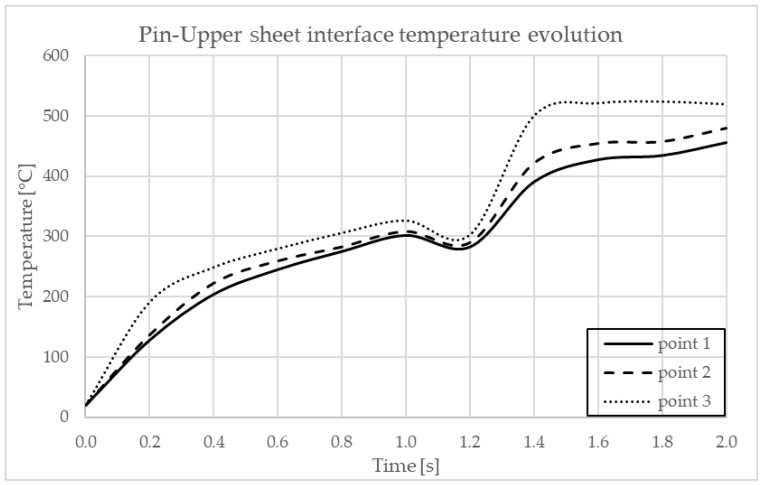
Temperature dynamics during FEA at the pin–upper sheet interface in AA7075-T6.

**Figure 14:**
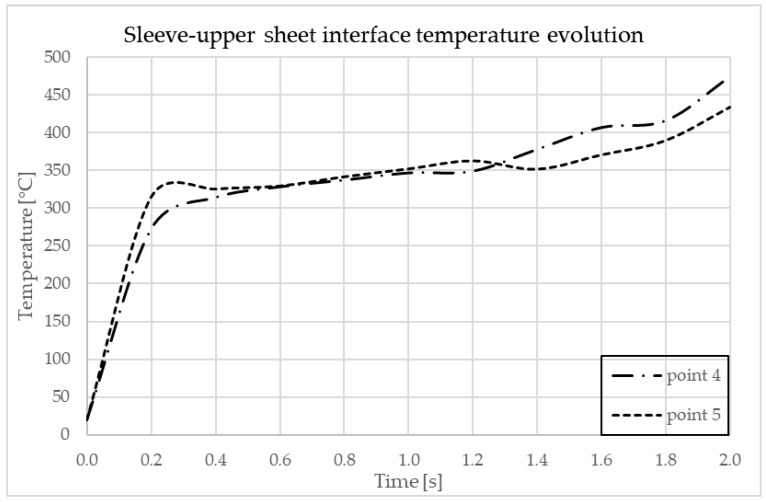
Temperature dynamics during FEA at the sleeve–upper sheet interface in AA7075-T6.

**Figure 15:**
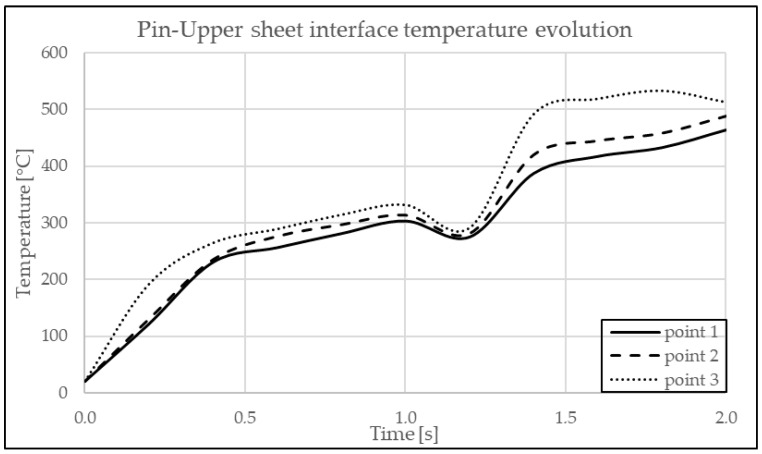
Temperature dynamics during FEA at the pin–upper sheet interface in AA6061-T6.

**Figure 16:**
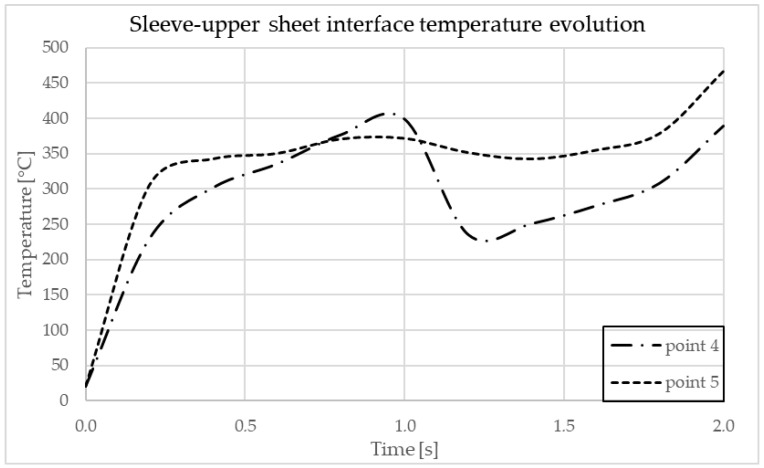
Temperature dynamics during FEA at the sleeve–upper sheet interface in AA6061-T6.

In Figure 12 are indicated the points where the temperature is observed. In Figure 13 and Figure 14, the graphs showing the temperature evolution over time at the points indicated in Figure 12 can be observed, at 3000 rpm and a configuration with the upper plate made of AA7075-T6 and the lower plate made of AA6061-T6; in Figure 15 and Figure 16, the graphs show the temperature evolution when the positioning of the two plates is reversed.

#### 3.1.2. Mechanical Behavior during RFSSW Process

**Figure 17:**
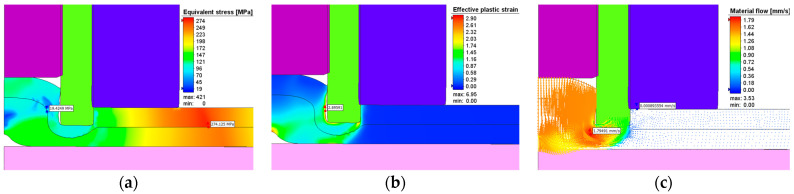
(**a**) Effective stress (MPa), (**b**) plastic deformation, and (**c**) material flow during the plunging stage in AA7075-T6.

**Figure 18:**
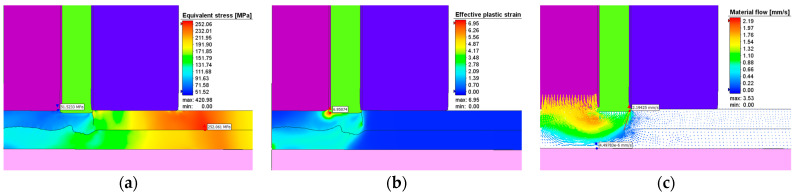
(**a**) Effective stress (MPa), (**b**) plastic deformation, and (**c**) material flow during the refilling stage in AA7075-T6.

**Figure 19:**
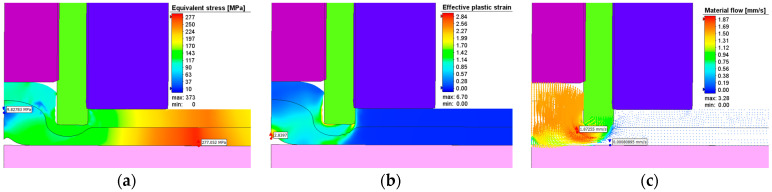
(**a**) Effective stress (MPa), (**b**) plastic deformation, and (**c**) material flow during the plunging stage in AA6061-T6.

**Figure 20:**
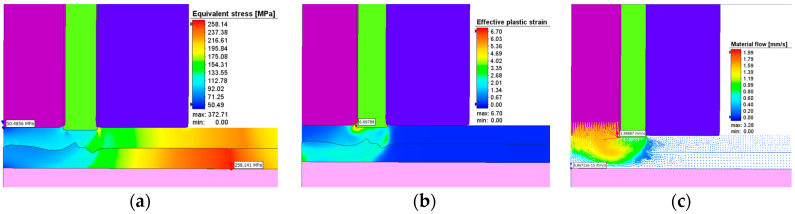
(**a**) Effective stress (MPa), (**b**) plastic deformation, and (**c**) material flow during the refilling stage in AA6061-T6.

The effective stresses, plastic strains, and material flow at one second after the start of the plunging stage can be seen in Figure 17 while Figure 18 show these at the end of the refilling stage. The same applies for Figure 19 and Figure 20. It can be seen that the maximum plastic strain reaches the value of 6.95 at the end of the welding process. The highest effective stress recorded is 421 MPa, located at the interface between the sleeve and the top plate at the beginning of the welding process before the aluminum is significantly heated by friction. The maximum residual effective stress is approximately 250 MPa, as shown in Figure 18a and Figure 20a.

The material flow vectors in Figure 17, Figure 18, Figure 19 and Figure 20c depict the flow patterns at the end of both welding phases. During the plunge phase, significant deformations occur in the aluminum plates near the pin area, as seen in Figure 17, Figure 18 and Figure 19. The material tends to flow in the direction of the pin, while compression is observed in the sleeve area.

Common defects in RFSSW joints include incomplete filling (Figure 8) and structural notches in the base material of the upper sheet. Incomplete refills may occur because the material, after undergoing plastic deformation, flows horizontally without dimensional constraints in that direction. Another factor might be the variation in material plasticity in this area compared with beneath the sleeve due to differing friction levels between the tool and the workpiece. The size of the structural notch is significantly influenced by the tool’s immersion depth, which, in turn, affects the joint’s load-bearing capacity. In the finite element model and the actual welded part for the parameters used, this type of defect was not observed, as shown in Figure 8.

### 3.2. Neural Networks Applied for Predictive Parameters Analysis

To assess the impact of welding parameters on the temperatures and stresses in the joint, an ANOVA analysis was conducted using neural networks.

Neural networks are mathematical models inspired by the workings of the biological nervous system. They are used to learn and generalize from input data so that they can make predictions or solve complex problems.

Neural networks are built based on the functioning of neurons in the human brain. Each neuron receives input signals, processes them, and produces an output signal. Similarly, in a neural network, we have nodes (artificial neurons) that receive input data, apply weights and activation functions, and generate output data.

Neural networks can learn from given examples. They are trained using a set of known input and output data. By adjusting the weights of connections between neurons, the network learns to make predictions or classify new data.

Once trained, neural networks can synthesize mathematical models to solve various problems.

Neural networks are utilized in numerous domains, such as speech recognition, natural language processing, medicine, finance, and more. Their capability to learn from examples provides substantial potential for application, particularly in solving complex problems [36,37,38]. By supplying a consistent set of examples and applying a rule to adjust the interneuron weights, the network can compare the computed output values with those provided by the examples, simultaneously adjusting the weights of the input data based on a predefined strategy. These features make neural networks highly suitable for ANOVA-type analyses. To conduct the mentioned analysis, RFSSW welding processes were simulated, varying the following welding regime parameters:

1. Retention time: It was changed in steps of 0.1 s, in the interval 1 ÷ 1.2 s, obtaining 3 situations.
2. Pin penetration depth: This has been modified according to the values of 0.8, 0.85, 0.9, and 0.95 mm.
3. Pin rotation speed: This was varied in the range of 2400 ÷ 3200 rpm, in steps of 200 rpm, obtaining 5 distinct situations.
4. The relative position of the two materials: Two situations were considered. In the first situation, the material with higher hardness was positioned at the top so that it came into contact with the pin, while in the second situation, the material with lower hardness was positioned at the top.

**Figure 21:**
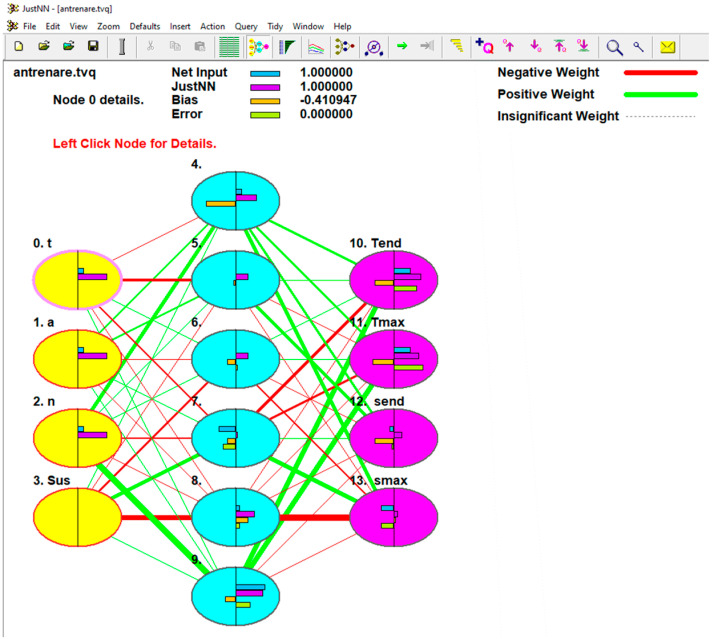
Neural network model for RFSSW process optimization.

A neural network was constructed and trained using 114 out of 120 examples, with the remaining 6 used to validate the results. The examples were generated by varying four parameters: holding times, penetration depths, rotation speeds, and relative positions of the materials. The output data included the maximum temperatures, temperature at the end of welding, maximum normal stress, and normal stress at the process conclusion. The neural network was built using JustNN version 4a, which does not allow for changes to the default network formation algorithm. The network was trained by importing data and adjusting values within specified limits until the output data set closely matched the original set. The final neural network featured a single hidden layer, determined by the formula *n* = √(*i∙o*) where i and o represent the number of input and output variables, respectively. In this case, both the input and output variables totaled four, suggesting the use of four hidden layers. However, only one hidden layer was ultimately utilized in the neural network. Further testing revealed that the training time was excessively long and the improvement in prediction accuracy was minimal. Therefore, a network with a single hidden layer was chosen. Figure 21 provides a graphical representation of the neural network.

The network training control elements were maintained at JustNN’s default settings: a learning rate of 0.6, momentum of 0.8, initial validation after 100 cycles, and subsequent validations every 100 cycles. The training process was set to terminate when all errors fell below 0.01, indicating the absolute differences between the initial input values and the network-predicted values were within this margin. Upon completing the training, the impact of each input variable on the output variables was assessed. The variables, ranked by importance, were pin speed (*n*) with a score of 10.5081, material position with a score of 8.6151, pin penetration depth (*a*) with a score of 3.9364, and holding time (*t*) with a score of 3.8179.

**Figure 22:**
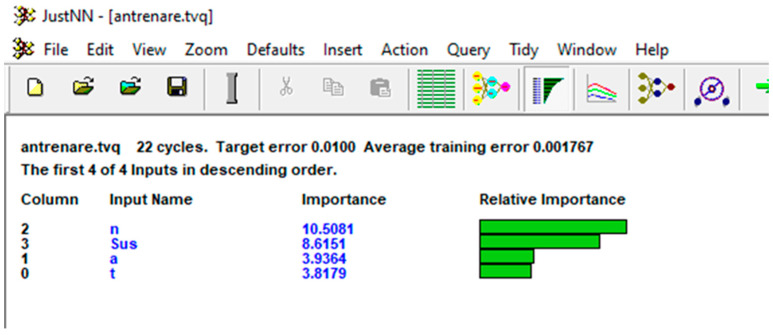
The NN model input parameters’ relative importance.

The importance of the inputs is the sum of the absolute weights of the connections from the input nodes to all nodes in the first hidden layer, see Figure 22. In other words, the more weight a connection has, the more important it is to the neural network. Entries are ordered in descending order of absolute importance. Thus, it starts with the most significant entry.

In general, this visualization helps us understand which input variables are most relevant to the predictions or results obtained by the neural network. It is a valuable tool in the process of analyzing and interpreting the functioning of neural networks.

**Figure 23:**
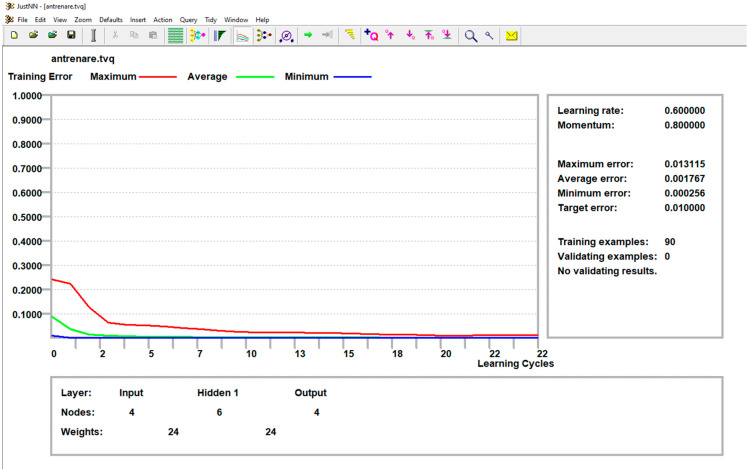
Neural network training error.

Figure 23 shows the graph produced at the end of the neural network training, depicting the learning curves for the maximum, average, and minimum errors, along with the maximum allowable error, set at 0.01. To verify and validate the network’s performance, six queries were conducted using data reserved for validation, which was not used during the training phase.

The results from these queries were compared with the simulation results, and the findings are summarized in Table 5.

The meaning of the parameters in Table 5 is as follows: 

*T_end_*—the temperature at the end of the welding process; 

*T_max_*—the maximum temperature reached during the welding process; 

*σ_end_*—the stress reached at the end of the process; 

*σ_max_*—the maximum stress reached during the process; 

*T_end calc_*—temperatures at the end of the welding process, predicted by querying the neural network; 

*T_max calc_*—the maximum temperature reached during the welding process as predicted by the neural network query; *σ_end calc_*—the stress at the end of the process as calculated by the neural network; *σ_max_*—the maximum stress during the process as predicted by the neural network query.

The relative error was calculated, for each of the output parameters, with the formula: $∆V=\frac{V_{s}-V_{c}}{V_{s}}\cdot 100$ [%], *Vs* being the value of each output parameter (*T_end_*, *T_max_*, *σ_end_*, *σ_max_*) obtained by modeling and *V_C_* the value of the same parameter obtained by querying the network.

We observe that the values obtained by querying the network are very close to those obtained by simulation.

This allows us to state that the neural network can predict the values of temperatures and tensions as a function of specific parameters, such as pin penetration, rotational speed, holding time, and position of dissimilar materials. A major advantage of using neural networks is the shorter response time and lower computational resources compared with traditional simulations. However, it should be kept in mind that the network predictions are realistic only within its training range, and the input values do not necessarily have to be equal to those in the training set.

## 4. Conclusions

The results of this study show that with the Simufact Forming software, it is possible to predict the stress and strain state during the RFSSW welding process of dissimilar aluminum alloy sheets. It is also possible to choose the optimal combination of welding parameters to achieve a joint with the lowest residual stress state.

During the welding process, there are two distinct phases: penetration and retraction. In the penetration phase, the temperature in the welding zone increases rapidly at a rate of 310 °C/s. This rapid increase in temperature facilitates the fusion of the material and the formation of a solid joint.

After the penetration phase, the withdrawal phase follows. In this step, the temperature initially decreases, but then increases again at a rate of about 700 °C/s for a period of 0.3 s. Under these conditions, the maximum temperature reaches 497 °C, ensuring a solid and durable weld.

The evolution of the temperature over time is essential to ensuring the quality of the weld. Both experimental data provided by thermocouples and finite element analysis were used to track the temperature evolution. The results obtained by these two methods are very similar, with a data correlation rate of over 90%.

Regarding the stresses in the parts subjected to the welding process and the material flow in the joint area, they are very similar in value for both experimental and simulation data. This suggests that the simulation process is consistent and produces reliable results.

Welding parameters play an important role in determining weld quality. In descending order of importance, they are pin speed (*n*), the position of the two materials, pin penetration depth (*a*), and hold time (*t*).

Trained neural networks can be used to predict temperature and stress values under specific conditions of pin penetration, material position, rotation speed, and hold time.

Regarding future research directions, we propose to focus on the study of grain size evolution in friction stir welding (RFSSW). This modern welding process has the potential to open up new opportunities in manufacturing.

## Figures and Tables

**Table 1:** Chemical composition of the aluminum alloys (min–max%).

	Al	Mg	Si	Fe	Cu	Cr	Zn	Ti	Mn
AA7075	87.1 91.4	2.1 2.9	0 0.4	0 0.5	1.2 2.0	0.18 0.28	5.1 6.1	0 0.2	0 0.3
AA6061	95.8 98.6	0.8 1.2	0.4 0.8	<= 0.7	0.15 0.4	0.04 0.35	<= 0.25	<= 0.15	<= 0.15

**Table 2:** Parameters used to realize the joints in Figure 2.

Point	Rotation Speed [rpm]	Penetration Depth [mm]	Point	Rotation Speed [rpm]	Penetration Depth [mm]	Point	Rotation Speed [rpm]	Penetration Depth [mm]
1	2400	0.8	7	2400	0.85	13	2400	0.9
2	2600	0.8	8	2600	0.85	14	2600	0.9
3	2800	0.8	9	2800	0.85	15	2800	0.9
4	3000	0.8	10	3000	0.85	16	3000	0.9
5	3200	0.8	11	3200	0.85	17	3200	0.9

**Table 3:** Basic mechanical properties of materials.

Material	Yield Stress Rp_0.2_ (MPa)	Ultimate Tensile Stress *Rm* (MPa)	Elongation at Fracture *A*, %	Melting Point °C
6061-T6 AA	276	310	12	582–652
7075-T6 AA	502	572	13	477–735

**Table 4:** Parameters in the plasticity model of base metal.

Mat.	*C*_1_	*C*_2_	*l*_1_	*l*_2_	*m*_1_	*m*_2_	*n*_1_	*n*_2_
AA 6061-T6	405.9	−0.0032	−4.344 × 10^−5^	0.01674	3.731 × 10^−4^	−0.05181	−5.610 × 10^−5^	0.2530
AA 7075-T6	506.5	−0.0044	5.756 × 10^−5^	−0.0280	2.659 × 10^−4^	−0.00110	−4.818 × 10^−5^	−0.1061

**Table 5:** Comparison of the output values provided by the neural network with those obtained through simulation.

*T_end_*	*T_end calc_*	Δ *T_end_* [%]	*T_max_*	*T_max calc_*	Δ*T_max_* [%]	*σ_end_*	*σ_end calc_*	Δ*σ_end_* [%]	*σ_max_*	*σ_max calc_*	Δ*σ_max_* [%]
559	562.63	0.65	582	582.78	0.13	261	256.11	1.87	373	371	0.54
554	556.57	0.46	568	570.36	0.42	267	264.1	1.09	416	420.84	1.16
443.5	450.1	1.49	462	468.79	1.47	257	255.59	0.55	416.5	418.79	0.55
459	459.24	0.05	483	483.09	0.02	254.5	253.03	0.58	371.5	371.53	0.01
510	511.32	0.26	525	523.55	0.28	256	255.95	0.02	421	419.54	0.35
523	530.72	1.48	545	552.17	1.32	253	253.26	0.10	374	372.64	0.36

## Data Availability

Data are contained within the article.
